# Non-Redundant Unique Interface Structures as Templates for Modeling Protein Interactions

**DOI:** 10.1371/journal.pone.0086738

**Published:** 2014-01-27

**Authors:** Engin Cukuroglu, Attila Gursoy, Ruth Nussinov, Ozlem Keskin

**Affiliations:** 1 Center for Computational Biology and Bioinformatics and College of Engineering, Koc University, Istanbul, Turkey; 2 National Cancer Institute, Cancer and Inflammation Program, Frederick National Laboratory for Cancer Research, Leidos Biomedical Research, Inc., National Cancer Institute, Frederick, Maryland, United States of America; 3 Sackler Institute of Molecular Medicine, Department of Human Genetics and Molecular Medicine, Sackler School of Medicine, Tel Aviv University, Tel Aviv, Israel; Institute for Research in Biomedicine, Spain

## Abstract

Improvements in experimental techniques increasingly provide structural data relating to protein-protein interactions. Classification of structural details of protein-protein interactions can provide valuable insights for modeling and abstracting design principles. Here, we aim to cluster protein-protein interactions by their interface structures, and to exploit these clusters to obtain and study shared and distinct protein binding sites. We find that there are 22604 unique interface structures in the PDB. These unique interfaces, which provide a rich resource of structural data of protein-protein interactions, can be used for template-based docking. We test the specificity of these non-redundant unique interface structures by finding protein pairs which have multiple binding sites. We suggest that residues with more than 40% relative accessible surface area should be considered as surface residues in template-based docking studies. This comprehensive study of protein interface structures can serve as a resource for the community. The dataset can be accessed at http://prism.ccbb.ku.edu.tr/piface.

## Introduction

Proteins physically interact with each other through their binding sites. Some proteins interact with their partners simultaneously using different interaction sites, some interact with their partners via the same interaction site at different times, and some appear to interact with only one protein [1]. How can many different proteins use the same binding interface, and how can a single protein bind many different proteins at the same time, are key questions that emerge in structurally-enriched protein-protein interaction networks and regulation. Within the framework of the general factors that bear on these intriguing questions is the landscape of residue conformations, particularly of key residues, making multiple and simultaneous interactions possible [2]–[7]. While a vast number of protein-protein interactions can take place, there is a limited number of specific binding site conformations through which proteins can bind [8]–[10]. Studies of interfaces can be illuminating: they can address questions such as whether preferences of specific amino acids in certain positions can help binding site prediction, and on a different level, how some proteins can bind many different proteins using the same binding site conformations. Since binding and folding are similar events, they may also help understand hierarchical protein folding [11]. Obtaining a set of unique interface structures can be particularly useful in template-based docking [12], [13]. We previously showed that template based docking can be fast and accurate if there exists a good set of template interfaces [14], [15]. Kundrotas et al. posited that unique interface structures can serve as templates to model nearly all complexes of structurally characterized proteins, and that the existing interfaces already can achieve this aim [13]. Kundrotas and Vakser showed that structural similarities of the interfaces have the greatest influence in template-based docking [16].

The main step to achieve a unique interface set is to flag similar interfaces. Comparisons of two protein interfaces can detect similarities in amino acid sequences of protein interfaces (sequence alignment) or similarities in 3D coordinates of amino acid positions in the proteins (non-sequential structural alignment). Protein interface clustering can be done in three different ways: using only sequential or only structural alignment scores of all protein interfaces, or a hybrid strategy which includes both sequential and structural alignment scores of protein interfaces. The PFAM [17] and SCOP [18] databases are commonly used for classification by sequence and structural alignments, respectively. Previous studies aiming to investigate binding properties showed that protein interfaces can be classified by their sequence similarities [8], [19] or, in other words, proteins with similar sequences often interact in similar ways [20], [21]. However, it has also been suggested that interactions can only be reliably inferred for close homologs [20], [22]. To decrease the computational cost, studies have also used a hybrid strategy of both sequence and structural comparison, and to increase the reliability of the classification, others used structural alignment of protein interfaces [23]–[27]. Bordner and Gorin clustered biologically relevant interfaces with a hybrid strategy to provide a reliable catalog of evolutionary conserved protein-protein interfaces with a diverse set of properties [28].

Detecting evolutionarily related proteins via structural similarities is more reliable than via sequence similarities since structure is more conserved. Sequence based methods are easy to derive and computationally cheap, so sequence based methods are generally the starting point of the studies; however, this has two limitations [29]. First, proteins generally use interfacial areas in order to form their interactions which do not necessarily contain short local sequence motifs. Secondly, if the sequence similarity of two proteins falls below 40% it is hard to make any inference about their functions. Schroder and his colleagues show that even if structural comparison is computationally costlier than other methods it gives more reliable results [23]. In order to find structural similarity between two protein interfaces, the 3D coordinates of the atoms must be known. The number of deposited structures increases exponentially with improvements in experimental techniques. Thus, it should be possible to identify novel binding strategies (if any) by examining recent deposited structures. Our previous works showed that the number of distinct interface structures grows with the increasing number of PDB structures [2], [30], [31].

To classify protein interface structures by either sequence, or structural alignments, or both, some kind of clustering methods should be used. In most structural clustering studies, a hierarchical clustering algorithm is used. Ghoorah *et al.* compare structural alignment of interfaces by extracting dimensionless interface vectors using the center of mass of core and rim Cα coordinates [24]. Interface vectors are clustered using hierarchical clustering. Aloy and his colleagues classify domain based interactions using 3D structures of proteins and perform complete linkage hierarchical clustering to find global interfaces [32]. Tseng and Li generate Protein Surface Classification (PSC) library using pairwise local RMSD measures of protein surfaces [33]. They present 1974 surface types that include 25857 functional surfaces identified from 24170 bound structures. Also, Teyra *et al.* perform pairwise structural alignments of protein binding regions and classify them with agglomerative hierarchical clustering using the complete linkage method [34]. They also note that complete linkage is sensitive to zero similarity and expands the differences between the clusters.

There are methods other than hierarchical clustering, such as centroid models (k-means clustering), distribution models (multivariate normal distributions), density models, subspace models (biclustering), and graph based models. Graph theory has also started to become popular in the last decade for analyzing the relationship between events. Barabasi and his colleagues presented various properties of networks by tracking the internet routes [35]. They discovered the power law distribution of the networks and the importance of the most highly connected nodes in lethality and centrality [36]. Other researchers focus on topological properties of networks. Girvan and Newman highlighted the betweenness property (first proposed by Freeman [37]) of the nodes and edges of the network, and emphasized the community structure of the network [38]. Edge (node) betweenness is defined as the number of shortest paths passing through this edge (node) between all pairs of other nodes. It is a measure of the influence of edge (node) on the flow of information between other edges (nodes). If a network has communities, few edges which have higher betweenness connect these communities. Removing the edges with highest betweenness value from the network means separating the communities from one another [38]. Girvan and Newman presented this method to sidestep the shortcomings of the hierarchical clustering.

In light of previous structural classification studies and the exponentially increasing number of Protein Data Bank (PDB) [39] structures, we present a protein-protein interface clustering method which combines the structural alignments of the protein interfaces and graph theory properties in order to extract protein interface representatives in the PDB. We used the Girvan and Newman method to cluster similar interfaces.

The novelty in this work is in generating structurally non-redundant protein-protein interfaces which are sensitive to small perturbations in protein binding sites that have a significant impact in template-based docking. Template-based docking strategies are based on non-redundant structures of protein interfaces which are compared with target monomer surfaces aiming to match similar interface partners [12]. Here we constructed a set of unique interfaces from the PDB, and carried out a comprehensive analysis of this set with respect to three properties. The first property is the reliability of the new clusters. We generated different clustering criteria for finding the best clusters then compared our dataset with the previous dataset which was generated by using a hierarchical clustering method. The new clusters outperformed previous clusters. Secondly, we searched for the best method to extract surface residues of protein monomers for template-based docking using the new structurally non-redundant interface clusters. Third, we looked up proteins which interact with multiple partners in order to test the specificity of the non-redundant interface clusters. We also compared the protein interface clusters throughout the years.

22604 unique interface types are defined in our study. These can be exploited for template based docking, for studies of binding specificity, function-domain evolution, and drug design.

## Method

An interface is described as contact region between two interacting proteins. Previous works show that there are different approaches to find contact residues in a complex. Distance based approaches use atomic distances between two proteins to extract interacting residues [2], [30], [40]–[49], surface area based approaches use accessible surface area (ASA) values [50]–[53]. Some of the interfaces are derived by using Voronoi diagrams [54].

Two residues are defined as contacting if the distance between any two atoms of the two residues from different chains is less than the sum of their corresponding van der Waals radii plus 0.5 Å [2], [30]. We further define nearby residues if the distance between alpha carbon atoms of noninteracting residues and interacting residues in the same chain is smaller than 6 Å. Previous studies showed that nearby residues are important to represent a more complete architecture of interfaces, such that interface residues are not isolated [2], [9], [10], [30], [55].

An interface is labeled with the PDB ID plus the chain IDs. For instance, if the PDB ID of a protein structure is 1GQP and there is an interface between chains A and B, then this interface is named 1GQPAB as in our previous studies [2], [30], [31].

In order to generate a protein-protein interface dataset, we extract all possible binary interactions of the protein structures and check regardless of whether they interact. All PDB entries are used to generate the interface set. As of January 14^th^ 2012, there were 78477 PDB entries and 45491 of them were complex structures which resulted in 622321 possible binary interactions, assuming all protein chains interact with each other. However, not all chains in a protein complex interact physically with each other, necessitating chain pair detection. Extracting interface residues in complexes by distance thresholds needs excessive time. Hence, the accessible surface area (ASA) of the possible interface pairs are first calculated by using NACCESS [56] in order to eliminate interface candidates whose interface ASA values of the complex structure are smaller than 1 Å^2^. NACCESS calculates the monomer and complex ASA values of the proteins and the interaction surface is calculated simply subtracting complex ASA value from the monomer ASA values of two proteins. When small interfaces were eliminated, 184342 interface candidates remained. (For NMR structures the first model is used. RNA and DNA chains are eliminated. Chains which have residues different than usual 20 amino acids are eliminated (e.g. selenomethionin)). The remaining interface candidates were processed according to our interface definition. As a result, there were 130209 interfaces where each binding site had at least five residues.

### Structural Comparison

MultiProt [57] was used to compare these 130209 protein interfaces. MultiProt, which performs structural alignment regardless of the order of the residues on proteins, is an appropriate tool to use for comparison of interface structures which are generally composed of discontinuous segments of protein chains. No sequence alignment was performed. MultiProt uses PDB structures as input to calculate the binary similarities of each protein in the dataset. In each comparison step, two interface files which have contact and nearby residues in PDB format are given as an input to MultiProt in order to perform structural alignment.

### Interface Similarity

Interface similarity is calculated based on the structurally matched residues of the two interfaces. The similarity formula is:
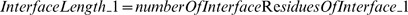


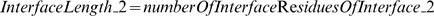
numberOfStructurally Residues are found by using MultiProt [57]. MultiProt aligns the interfaces structures using the geometric hashing algorithm. We used 3 Angstroms as the rmsd threshold in the structural comparisons.

The Interface similarity is defined as
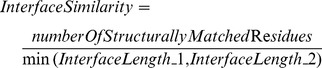
(1)


Pairwise comparison of all interfaces is a time consuming process. Comparing a small interface with a big interface is unnecessary. Gao and Skolnick’s [58] work on the structural space of protein interfaces shows that native interfaces find a match with a significant score among random interfaces and their mean interface residues coverage is 86% with a standard deviation 10%, and their mean contact residues coverage is 52% with a standard deviation 9%. Thus, to decrease the number of interface comparisons, we eliminated interface comparisons where the number of contacting residues in one interface is larger by 25% than that of the small interface and the number of contacting and nearby residues is larger by 50% as compared to the smaller interface. As a result, nearly 2 billion protein interface comparisons are done by MultiProt which used approximately 2500 cpu days to finish the comparisons.

### Clustering Algorithm

**Figure 1:**
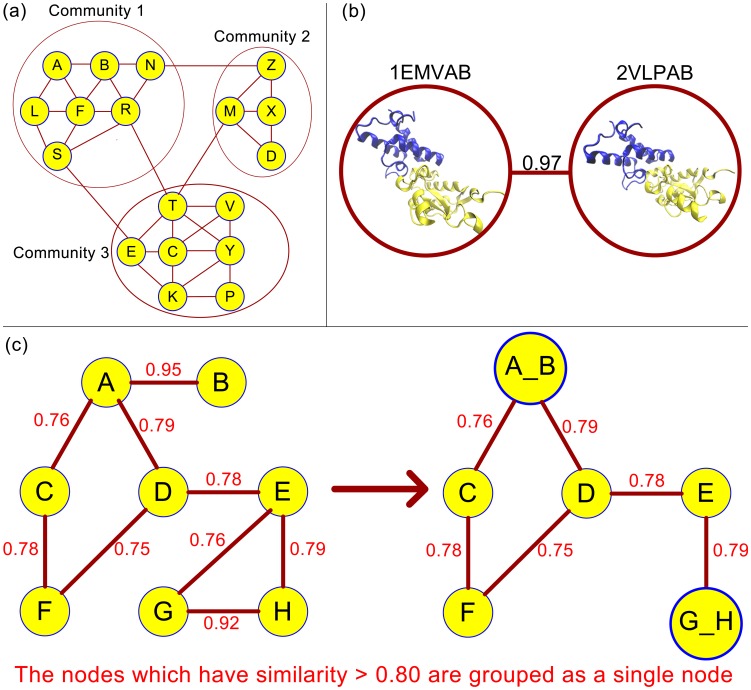
Network of Interfaces. (a) Community structure. (b) Node and edge representation in protein interface network. (c) The nodes in the left network which have similarity values higher than 0.80 are grouped as a single node in the right network. The new node and the neighbors’ similarity values are chosen as the maximum similarity value of the edges between the two nodes which were grouped and the neighbor nodes (e.g. Node G and node H).

Girvan and Newman [38] used graphs in order to find communities in a network. A community is defined by dense inter-connectivity (Fig. 1a). Communities are clusters in a network which have similar properties. Communities are extracted using the betweenness property of the edges. An edge between communities has the highest betweenness value in the graph. The method of Girvan and Newman starts with removing the edge with the highest betweenness value and then, recalculates all the betweenness values of the edges and removes the edge from the graph that has the new highest betweenness value. Communities in a network are found applying this process until the stopping criteria are reached. Therefore, similar protein interfaces can be clustered using community finding algorithm. In order to find the clusters of similar interfaces, the network of interfaces should be formed first.

#### Network of interfaces

A network is formed by nodes and edges. The network of protein interfaces is constructed by using interface structures as nodes. If two interfaces are similar (the similarity is calculated as explained above, Equation 1), an edge is drawn between the two corresponding nodes in the network (Fig. 1b). This differs from our earlier strategy of constructing the protein interface network [59] where protein monomers are considered as nodes. There, an edge is drawn if the two monomers form an interface. Here we represent each interface structure as a node and similarity between interface structures as edges.

#### Finding communities

Dividing a network structure to community structures starts with removing the edge which has the highest betweenness value. If the network is separated into two distinct networks, the edge removing process keeps going recursively for each network until one of the stopping criteria is reached. Stopping criteria are based on two network properties, clustering coefficient and minimum cluster size. The clustering coefficient is a measure of degree for nodes in a graph which tend to cluster together. The clustering coefficient [38] is calculated by
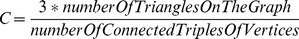
(2)


As a stopping criterion, different clustering coefficient values are used during clustering (1, 0.95, 0.90, 0.85 …, and 0.5) in order to find the best possible clusters. If the network has a clustering coefficient value higher than the criterion the network separation process stops.

Minimum cluster size criterion, set at 5, is used because we do not want network nodes to fall apart as a network of size 1. After a network separation, if one of the clusters has less than 5 interfaces, this cluster is no more divided into two clusters.

In order to increase the speed of network separation, the nodes in the network are clustered in 5 steps according to the interface similarity values (increasing similarity values with 5%, similarity values higher than 0.80, 0.85, 0.90, 0.95 and 1.00 are processed respectively). Nodes which have similarity values higher than 0.80 are grouped as a single node (starting with the nodes which have the highest similarity) and their neighbors are linked to this new node (Fig. 1c). The new node and the neighbors’ similarity values are chosen as the maximum similarity value of the edges between the two nodes which were grouped and the neighboring nodes. Conversely, the new node and the neighbors’ similarity values are chosen as the minimum similarity value of the edges between the two nodes which were grouped and the neighbor nodes. The final clustering results are better with the maximum value similarity (shown by validation of the clusters with different criteria in the Results section).

The new networks generated by the community finding algorithm are processed again, combining nodes which have similarity values higher than 0.85, 0.90, 0.95 and 1. After five cycles, all generated networks are reprocessed by the community finding algorithm without any node grouping to obtain the final results. Communities extracted at the final stage are the interface clusters.

**Figure 2:**
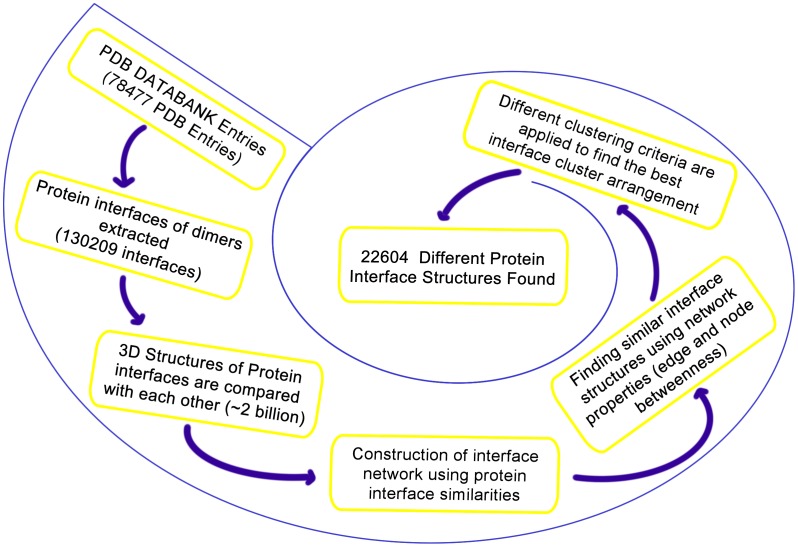
Flowchart of the methodology.

This grouping of the nodes according to their similarity values procedure is used to minimize the run time of the community finding algorithm. Calculating edge betweenness centrality of a network has O(n^3^) complexity. The largest connected component of the network has 1638090 edges which restrict applying the community finding algorithm because of the runtime. Edge betweenness centrality values of the new network should be calculated after each edge removing process, so this step is the bottleneck of the community finding algorithm. Therefore, the nodes are grouped according to their similarities, then the separation step is applied which speeds up the removal of the edges from the network, and simplifies finding the edge betweenness values of the network in the last step. The main steps of the methods are shown in Fig. 2.

### Evaluating Communities

The clustering results are evaluated using the Silhouette index [60]. The performance of the Silhouette index has been reviewed in Handl et al. [61]. The silhouette index is calculated as:
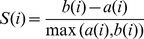
(3)where

a(i) = the average dissimilarity between interface i and all other interfaces in its cluster.

b(i) = the minimum of the average dissimilarities between interface i and the interfaces in the other clusters.

### Software

The clustering procedure was performed using an in-house software. Codes are written in Python, and the betweenness and clustering coefficient values are found by using the NetworkX package [62].

## Results and Discussion

### Validation of the Clusters with Different Criteria

There are various clustering methods and they have different pros and cons. In this work, we used two step approaches for a reliable clustering result. First, grouping the nodes according to their similarities iteratively is used to provide compactness (small intra-cluster variation) of the clusters. Secondly, the edge betweenness property of the protein interface network is used to cluster similar interfaces, providing the connectedness of the clusters.

Choosing the clustering method is the starting point of the data analysis but the important part is finding appropriate criteria for generating the best results. Different clusters are obtained by using different parameters and all results from different clusters are evaluated to select the best cluster set. The parameters for generating different clusters are the clustering coefficient for the stopping criteria, the similarity value adjustments (increasing similarity values by 5% or 1% for grouping similar interfaces as a single node), and choosing the maximum or minimum similarity values for the new node and the neighboring nodes during node formation.

**Figure 3:**
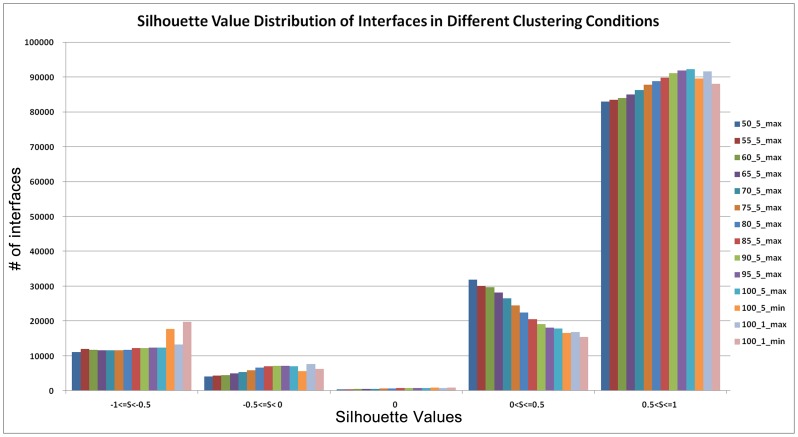
Silhouette value distribution of interfaces in different clustering conditions. For example 60_5_max means that clustering coefficient for stopping criteria is 0.6, similarity increment for grouping is 5 and maximum similarity value is used for edges from the combined node to their neighbor nodes.

For the evaluation of a clustering method, there are two main measurements; external and internal [61]. For external evaluation, we need a gold standard protein interface clusters dataset from the PDB, but unfortunately there are no standard clusters for protein interfaces. For internal measurement, we used Silhouette index [60] for comparing different clusters generated by different thresholds such as various clustering coefficient values for stopping criteria (1, 0.95, 0.90, …, 0.50) and different similarity values for grouping the nodes (1% or 5% increment of similarity value for grouping the similar interfaces). As a result, the silhouette index shows that using 1 as a clustering coefficient, 5% increment of similarity value for grouping similar interfaces, and choosing the maximum similarity value between the combined nodes and neighbor node gives the best protein-protein interface clustering results. In Fig. 3, the silhouette index for 100_5_max bar (clustering coefficient of 1, 5% increment of similarity value for grouping similar interfaces are chosen, and the maximum similarity value between the combined nodes and neighbor node is selected) gives the best silhouette values. This corresponds to 22604 clusters and 11088 of these clusters are single-membered. We used these 22604 clusters for further analysis which included both single and multi-membered clusters.

**Figure 4:**
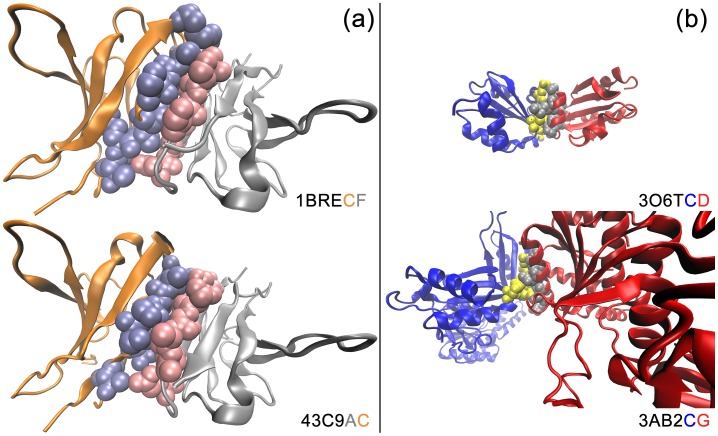
Similar interfaces with similar and dissimilar global protein folds. Complexes are shown in cartoon representation and interface residues are shown in ball representation. (a) Bence-Jones Kappa I Protein Bre complex (1BRECF) and Immunoglobulin Light and Heavy chain complex (43C9AC) have 79% interface similarity. (b) Asportakinase complex (3AB2CG) and Thioredoxin complex (3O6TCD) which have different global fold have 77% interface similarity.

Investigation of the interface clusters reveals that as expected, protein interactions can occur between homo- or heterodimeric chains. Further, when members of a single interface cluster are investigated, different types of interfaces are observed. We previously labeled three interface types: Type 1: *Similar interfaces, similar global protein folds*. In most cases, if the interfaces are similar, the overall protein folds are also similar. Such similar interface, similar fold clusters contain a single family. Here, we observe that (as also expected), protein interface structures can be similar when the global folds of the complexes are similar. An example is shown in Fig. 4a. Bence-Jones Kappa I Protein Bre complex (1BRECF) and Immunoglobulin Light and Heavy chain complex (43C9AC) have 79% interface similarity as shown in the figure. Type 2: *Similar interfaces, dissimilar global protein folds.* Even if the global folds of proteins are different, these proteins can interact by similar interface structures as shown in Fig. 4b. Asportakinase complex (3AB2CG) and Thioredoxin complex (3O6TCD) which have different global folds have 77% interface similarity as shown in the figure.

### Comparison with Previous Dataset

**Figure 5:**
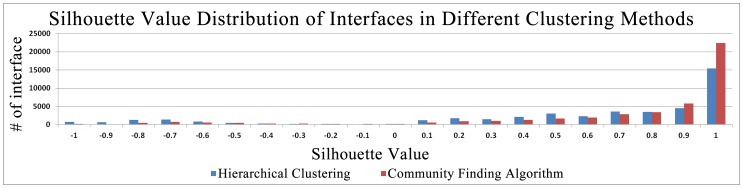
Comparison of the silhouette values. The silhouette value distribution over the protein interfaces generated by hierarchical clustering and community finding algorithm is presented. The new clusters are better clustered according to the silhouette values.

In order to show the performance of the current dataset, we compared it with our previously derived non-redundant protein interface dataset [31]. Our previous dataset is derived by using hierarchical clustering method and the important innovation of the current dataset is replacing the hierarchical clustering algorithm with a community finding algorithm based on graph theory. Further, the previous clustering operated with the requirement that the maximum interface size difference between cluster members should be maximum fifty residues; the current clustering method uses a percentage-based approach. There will be similarity between the interfaces if the bigger interface size does not exceed the 1.25 times of the smaller interface size. This strategy also outperformed with smaller interfaces. Hence, the new method extracted different interface structures than the previous published method. 45176 interface structures are clustered in our previous dataset and 7240 unique interface structures were found. The new clustering algorithm is applied to these 45176 interface structures and 8648 unique interface structures are obtained. When their silhouette values are examined, the previous clusters overall silhouette value is 0.52 and the new clusters overall silhouette value is 0.70. The distribution of silhouette values over the number of interfaces explicitly showed that the community finding algorithm based on graph theory outperformed the hierarchical clustering (Fig. 5).

We also tried to compare our new dataset with the work of Kim W.K. *et al.* [23]. The benchmark data used in their work is generated by using domain information of the complexes. In our case, we investigated interactions between protein monomers. Therefore, in our protein-protein interactions there are multi-domain interactions at the same time. For example, in the benchmark set, the interface between the chain A and B of PDB ID “1A22” exists in two different clusters because chain B has two SCOP domains. The SCOP domain in Chain A interacts with two different SCOP domains in Chain B. However, in our dataset, we labeled these three domain interactions as one interface structure. Multiple domain entries always cause problems in comparison. Another problem is the difference between the minimum number of residue interactions in order to form an interface structure. An interface should have at least 5 interacting residues in each monomer according to our definition, however, in the benchmark, some domain-domain interactions have less than 5 interacting residues in the monomer. Therefore, we could not compare our results with the benchmark dataset of Kim et al.

### Surface Extraction of Monomers for Template Based Docking

**Figure 6:**
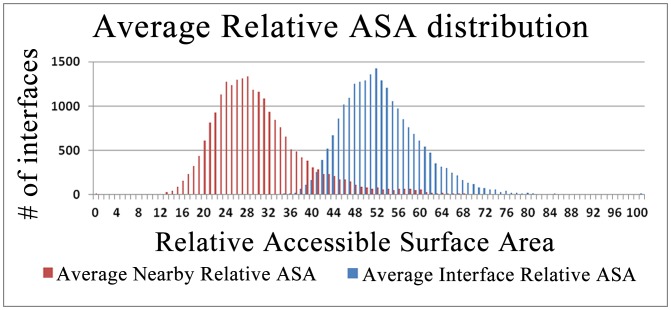
Comparison of the average relative accessible surface areas. The average relative accessible surface area of the representative interfaces and their nearby residues are showed. We suggested using 40% RASA value which corresponded to 99% of the average interface RASA values in order to extract interface residues using RASA values of the residues.

Structurally non-redundant interface architectures are obtained by extracting representatives of all clusters. These representative interfaces are a valuable resource for template based docking studies. The main purpose of template based docking is matching monomer surface to a template interface structure; thus surface extraction of the monomers is an important step. In order to obtain reliable docking results, the same method should be used to extract the surface of monomers with the templates. Jones and Thornton [63] used relative accessible area (RASA) of the residues to define the interior and exterior residues. They defined exterior residues as having RASA value >5%, and interior residues as those with RASA value < = 5%. However, when we analyze the distribution of the RASA values over the interface and its nearby residues, we found that the mean of the average RASA values of each representative interface residues was 52.58 with a standard deviation of 7.84. The mean of the average RASA values of each representative nearby residues was 29.72 with a standard deviation of 9.12. Hence, we suggested using 40% RASA value which corresponded to 99% of the average interface RASA values in order to extract interface residues using RASA values of the residues. Both interface and nearby residue distributions of representative interfaces are shown in Fig. 6.

### Sensitivity of the Templates: Multi-interface Binding Strategy for the same Protein Pairs

A protein can interact with its partners using the same or different interfaces. Finding possible interaction sites is challenging and it is critical to predict possible interaction modes. Docking, homology modeling, and template based docking can be used for this purpose. When all PDB entries are investigated, some proteins with multiple partners are observed to exploit multiple interaction sites with their partners. These experimentally found structures can be extracted using distinct interface structures of the complexes (representative interface structures) present in PDB.

**Figure 7:**
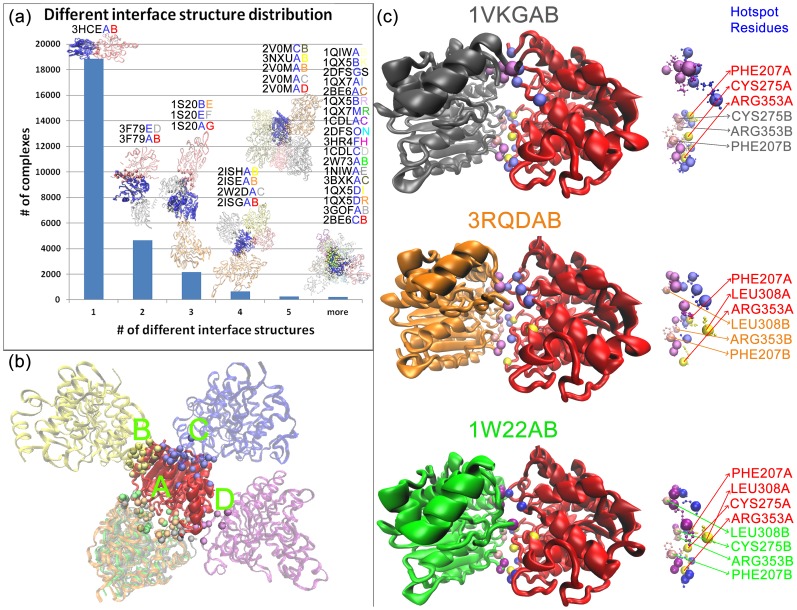
Multi-interface binding strategy for the same protein pairs. (**a**) Histogram of protein-protein interactions which have different binding structures at the same shared site or different binding sites. 7962 protein-protein pairwise interactions use more than one interface conformation in order to interact with the same partner. Complex with one interface structure is pair of Phenylethanolamine N-methyltransferase with Phenylethanolamine N-methyltransferase, with two interface structures are pairs of probable two-component response regulator with probable two-component response regulator, with three interface structures are pairs of Hypothetical oxidoreductase yiaK with Hypothetical oxidoreductase yiaK, with four interface structures are pairs of Neurotoxin BoNT/A with Neurotoxin BoNT/A, with five different interface structures are pairs of Cytochrome P450 3A4 with Cytochrome P450 3A4 and eighteen interface structures are pairs of Calmodulin 2 with Calmodulin 2. (**b**) Multiple interaction sites of Histone deacetylase 8 (red-1VKGA). Six different interface architectures of interaction between Histone deacetylase 8 and Histone deacetylase 8 are shown. One of the binding sites is shared by three partners. The others are different. Gray-1VKGB, orange-3RQDB, green-1W22B are at binding site A, yellow-3F0RC is at binding site B, blue-3F07B is at binding site C, purple-1T64B is at binding site D. The balls represent the carbon alpha atoms of the interface residues of the complexes. Carbon alpha atoms are labeled according to interaction partners of the 1VKGA. (**c**) Histone deacetylase 8 (red monomer) uses the same binding site to bind different partners. Small conformational changes in the interface residues assist binding the partners. On the left hand-side, protein complexes are shown and in the center, interface structures are shown in ball and stick. Blue and yellow balls are the interface residues of histone deacetylase 8 (red), and yellow balls also showed the hotspot residues of the interface. Pink and purple balls are the interface residues of the partner monomer shown in gray, orange and green, and pink balls also show the hotspot residues of the interface. In the right side, hotspot residues of the interfaces are given.

To find proteins with multiple interfaces, firstly, pairwise sequence alignments of all the monomers in the PDB are downloaded from PDB FTP Services (22 November 2012) and labeled with a cluster ID. All monomers which have the same cluster ID have 100% sequence identity. This leads to 48669 different cluster IDs. Then, two monomers which have an interface are labeled as first monomer cluster ID underscore second monomer cluster ID (e.g. 423_1002). According to their monomer sequence identities, 26825 distinct interface pairs are extracted. These pairs are compared with the structural clusters extracted from interface similarity. 7962 protein pairwise interactions out of 26825 have more than one interface in the PDB. A general view of the database is shown in Fig. 7a.

Proteins prefer different conformations to bind other proteins. Analysis of those 7962 monomer pairs illustrates that these proteins interact with their partners using slightly different conformations to bind their partners at the same shared site or at different binding sites. 3500 out of the 7962 use the same shared site to bind their partners and the rest employ different binding sites. For example, in Fig. 7b, the red labeled monomer, which is a histone deacetylase 8, is shown with multiple interaction partners at four different binding sites (a shared site and three different binding sites). Histone deacetylase 8 (gray, orange, green, yellow, blue, and purple) can interact with other histone deacetylase 8. It can pair up with another histone deacetylase 8 at four different binding sites, dependent on cellular conditions, phosphorylation states and mutations. Interestingly, these histone deacetylase 8 pairs (taken from the protein interface clusters with their properties listed in Table 1) exist in six different interface architectures.

**Table 1:** Six different interfaces for interaction of histone deacetylase 8 with another histone deacetylase 8 show that both at the same and different locations, the interface size varies across binding sites.

InterfaceName	Interface ASA (Å^2^)	# of interfaceresidue	Binding Site
1VKGAB	1152.59	25	A
3RQDAB	907.84	20	A
1W22AB	1089.86	24	A
3F0RAC	753.88	19	B
3F07CB	1076.17	21	C
1T64AB	423.46	10	D

Analysis of Fig. 7b illustrates that the binding site of histone deacetylase 8 is used multiple times; however, as can be seen in Fig. 7c these have different interface architectures (these interfaces are in different interface clusters because their interface similarities are lower than 75% similarity. The rmsd scores of the pairwise structural alignments of the complexes (over 672 aligned residues) generated by PDBeFOLD [64] are as follows: 1W22AB-1VKGAB:1.126 Å, 1W22AB-3RQDAB:2.305 Å and 1VKGAB-3RQDAB:1.756 Å (RMSD values are calculated for alpha carbons)). Thus, despite the similarity of monomers and binding sites, small conformational changes in the binding sites provide different interface architectures (like interologs [65]) and different relative energy distributions among the residues (Fig. 7c) [10]. The energy distributions of the residues are extracted using the HotRegion server [66]. HotRegion gives information about hotspot residues which contribute more to binding energy [67]–[69]. Hence, generating templates using sequence similarity is not a good choice for docking. On the other hand, templates prepared with interface structure similarities are sensitive leading to more reliable docking.

### Protein Interfaces and Interface Clusters based on Years

**Figure 8:**
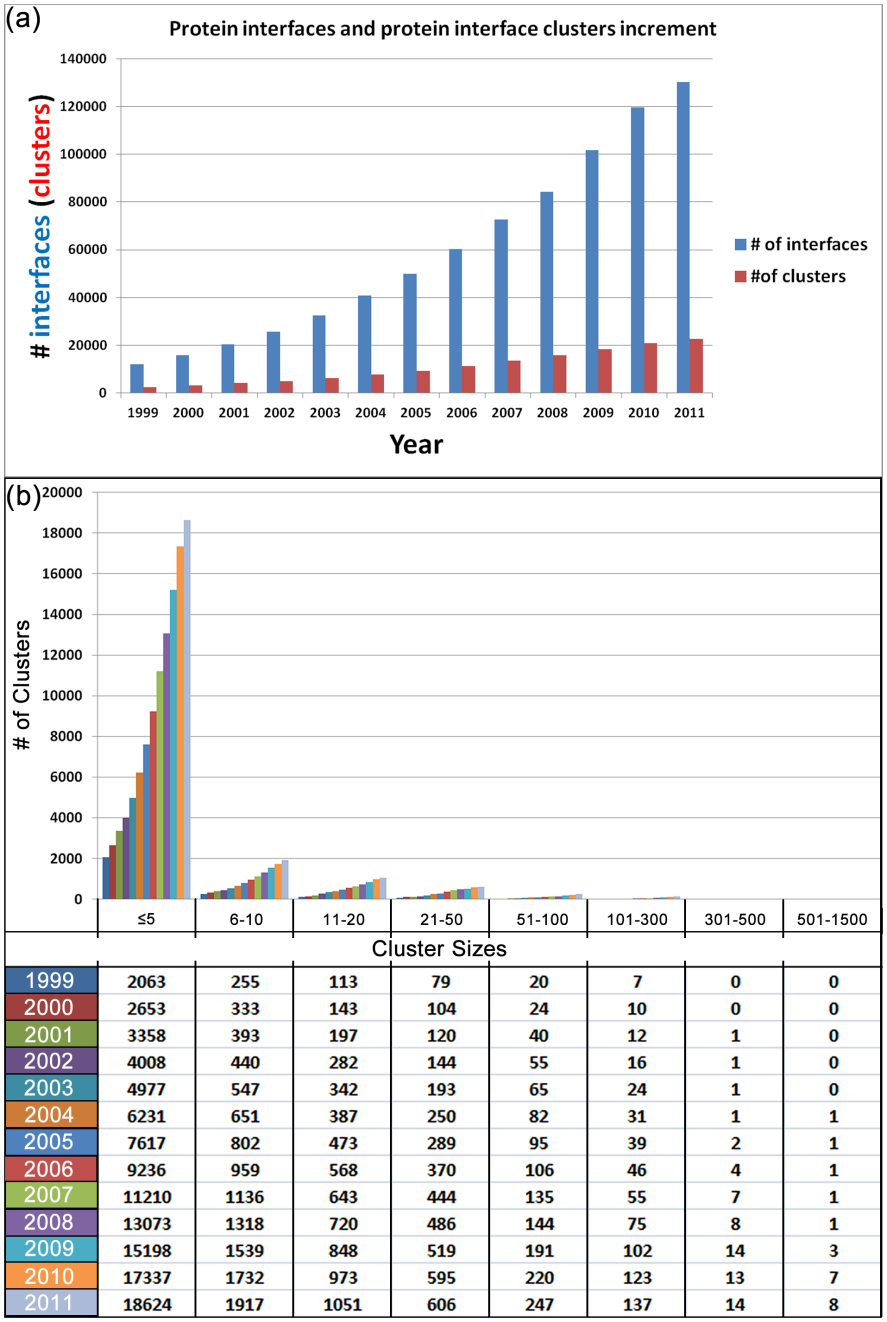
Protein interfaces and interface clusters based on years. (a) Protein interface and interface clusters evaluation during years. (b) Distribution of protein interface cluster sizes throughout years. While the number of protein interface clusters is increasing, the cluster sizes are getting denser. The largest cluster in 1999 had 238 members that increased to 1361 in 2011. The minimum cluster size criterion is used to stop the algorithm in order to prevent the network nodes as network size of 1. During separation of the networks, for example, if one of the networks divided into two networks which have 4 and 6 nodes respectively, the algorithm only tries to divide the network which is above 5 (if it is possible) because the other network reached its final state according to our stopping criterion.

The numbers of both available protein interfaces and their interface clusters increased exponentially in recent years. However, interface clusters have smaller increment than protein interfaces as shown in Fig. 8a. In addition to the increase in the number of clusters during the years, there is also an increase in the clusters size. The distribution of protein interface cluster sizes are presented in Fig. 8b. The largest cluster in 1999 had 238 members that increased to 1361 in 2011. Moreover, in 2011, there were 8 clusters with more than 500 members which were not present before 2004.

## Conclusions

Here we generate an updated non-redundant dataset of protein-protein interfaces, containing 22604 unique interface structures. The new dataset has not been used for prediction as yet; however, our older datasets have been extensively used for predictions of protein-protein interactions [14], [15], [70], [71]. In those works, we used subsets of our datasets, selected based on our aims, which were not as comprehensive as the full dataset. Protein-protein interactions reflect functional and structural information. This interface dataset can be analyzed with respect to all of these. The dataset is functionally unique in three ways: first, it provides a rich resource of structural data of protein-protein interactions, allowing using these for knowledge-based protein-protein interaction predictions, for constructing structural pathways, for studies of drug side effects, where drugs may bind to ‘unintended’ similar interfaces, for alternative pathways in drug resistance, and broadly for protein function. Second, analysis of these interfaces illustrates that 7962 of them are shared multi-partnered binding sites. Since the interfaces are derived from the PDB, it suggests that over one third of the protein-protein complexes have proteins bound to different partners. Some may be subunits; others may be signaling hub proteins. This allows dissecting protein-protein interactions to address questions such as how similar (or, different) are interfaces of partners binding to the same shared regions, on a large scale. We observe that interface size may vary substantially among partners, as do the hot spot residues in the interaction. Such shared binding site cases may help in addressing questions of binding specificity. And third, the analysis is performed with respect to interface residues. Extraction of the surface of monomers can be easily done by using RASA values of interface residues. Surface residues identified by the RASA values will work well with the current template set in template-based docking.

This dataset can be accessed at http://prism.ccbb.ku.edu.tr/piface.
